# Transcriptome analysis reveals that barnyard grass exudates increase the allelopathic potential of allelopathic and non-allelopathic rice (*Oryza sativa*) accessions

**DOI:** 10.1186/s12284-019-0290-1

**Published:** 2019-05-06

**Authors:** Qi Zhang, Xin-Yu Zheng, Shun-Xian Lin, Cheng-Zhen Gu, Li Li, Jia-Yu Li, Chang-Xun Fang, Hai-Bin He

**Affiliations:** Fujian Provincial Key Laboratory of Agroecological Processing and Safety Monitoring, College of Life Sciences, Fujian Agriculture and Forestry University, Cangshan District Shangxiadian Road No. 15, Fuzhou, 350002 China

**Keywords:** Rice (*Oryza sativa*), Allelopathy, RNA-Seq, Barnyard grass, Secondary metabolites, Induction

## Abstract

**Background:**

Allelopathy in rice (*Oryza sativa*) is a chemically induced response that is elevated by the exogenous application of chemical compounds and barnyard grass root exudates. An in-depth understanding of the response mechanisms of rice to chemical induction is necessary for the identification of target genes for increasing the allelopathic potential of rice. However, no previous studies have evaluated the transcriptomic changes associated with allelopathy in rice in response to barnyard grass exudates treatment. Thus, the aim of the present study was to reveal differentially expressed genes (DEGs) in allelopathic and non-allelopathic rice seedlings treated with barnyard grass exudates to identify target allelopathy genes.

**Results:**

The inhibitory effect of the culture solutions on the allelopathic rice accession PI312777 (PI) and the non-allelopathic rice accession Lemont (LE) significantly increased (*P* < 0.05) after treatment with barnyard grass root exudates. The RNA sequencing results revealed that 14,891 genes in PI(+B) vs. LE(+B), 12,505 genes in PI(+B) vs. PI(−B), and 5857 genes in LE(+B) vs. LE(−B) were differentially expressed following root exudates treatment. These DEGs were classified into three categories and 32 functional groups, i.e., 12 groups in the biological process category, 12 groups in the cellular component category, and eight groups in the molecular function category. There were 5857 and 2846 upregulated genes and 135 and 50 upregulated Gene Ontology terms (*P* < 0.05) in the biological process category in PI(+B) vs. PI(−B) and LE(+B) vs. LE(−B), respectively. These results indicated that the allelopathic accession PI is more sensitive than the non-allelopathic accession LE to exogenous root exudates treatment. Genes related to rice allelochemical-related biosynthesis pathways, particularly the shikimic acid and acetic acid pathways, were significantly differentially expressed in both rice accessions. These findings suggested that phenolic acids, fatty acids, and flavonoids, which constitute the downstream metabolites of the shikimic acid and acetic acid pathways, are significantly expressed in response to root exudates of barnyard grass.

**Conclusions:**

The allelopathic potential of both rice accessions could be significantly enhanced by barnyard grass root exudates application. Furthermore, genes related to the biosynthesis pathways of reported rice allelochemicals were significantly differentially expressed in both accessions. Phenylalanine ammonia lyase was determined to be a potential target for the regulation of chemical induction.

**Electronic supplementary material:**

The online version of this article (10.1186/s12284-019-0290-1) contains supplementary material, which is available to authorized users.

## Background

Since Dilday and his colleagues (Dilday et al. 1989) observed apparent allelopathic activity in certain rice (*Oryza sativa* L.) varieties in response to the aquatic weed ducksalad [*Heteranthera limosa* (Sw.) Wild] in field tests, there has been great interest in the application of rice allelopathy for controlling paddy weeds, thus reducing herbicide usage in rice production. However, three decades after Dilday’s study, rice allelopathy has yet to be widely applied in practice. The main reason is that rice allelopathy is a quantitative trait that is heavily influenced by environmental conditions (Dilday et al. 2000; Shin et al. 2000; Fang et al. 2010; Kato-Noguchi 2011; Wang et al. 2010; He et al. 2012). Rice allelopathic activity is polygenically controlled (Ebana et al. 2001; Jensen et al. 2001, 2008; He et al. 2004; Junaedi et al. 2007). To date, no candidate gene has been used for the genetic breeding of allelopathic rice cultivars. Although only 3–5% of screened rice accessions have been found to certain degree of allelopathic effect on one or more weed species (Dilday et al. 2000), Olofsdotter (1998) suggested that allelopathic genes are also present in modern varieties of rice. However, the high allelopathic activity of wild types may be reduced or lost in crops during hybridization and selection for other characteristics (Putnam and Duke 1974; Dilday et al. 2000). Studies have shown that abiotic stress, such as nitrogen (N), phosphorous (P), and potassium (K) deficiency (He et al. 2008; Wang et al. 2010; Fang et al. 2010) and UV-B irradiation (Shin et al. 2000), and biotic stress, such as the co-cultivation of rice and barnyard grass (Kato-Noguchi 2011; He et al. 2012), could enhance the allelopathic potential of allelopathic rice accessions. Recent studies show that allelopathy can be induced by exogenous application of chemicals, such as exogenous phenolic acids, methyl jasmonate, and methyl salicylate (Kong et al. 2004a; Bi et al. 2007; Fang et al. 2009; Xu et al. 2010; Kato-Noguchi 2011). Our previous study confirmed that rice allelopathy in both an allelopathic accession PI312777 (PI) and a non-allelopathic accession Lemont (LE) can be effectively elevated by treatment with barnyard grass root exudates in a hydroponic system (Zhang et al. 2018). Therefore, an in-depth understanding of chemical-induced mechanism of rice allelopathy is necessary for identification of target genes to increase rice allelopathic potential.

RNA sequencing (RNA-Seq) is an approach used to deduce and quantify transcriptomes (Wang et al. 2009) and quantitatively determine the RNA expression levels of transcripts under different conditions. RNA-Seq has recently been used to track gene expression changes in rice in response to abiotic stress, including zinc, P, and N deficiency, anoxia, and drought (Nguyen et al. 2013; Yang et al. 2015; Nanda et al. 2017; Deng et al. 2018), high temperature and cold stress (Shen et al. 2014; Dametto et al. 2015; Mangrauthia et al. 2018), drought and salinity stress (Shankar et al. 2016; Zhou et al. 2016; Zhang et al. 2016), cadmium and arsenic stress (Chakrabarty et al. 2009; He et al. 2015), as well as the characteristics of rice root heterosis and crown tissue under different conditions (Zhai et al. 2013; Nanda et al. 2017). However, no studies have evaluated the transcriptomic changes in allelopathy in response to chemical induction in rice. Previous studies showed that rice accessions expressed allelopathic effect on barnyard grass (*Echinochloa crus-galli*) at 3–7 leaf stages. (Hassan et al. 1998; Li et al. 2015). Vergara (1992) reported that rice yield was not significantly affected when paddy weeds were removed within 30 days after transplantation. Increasing rice allelopathic potential as early as possible can enhance the ability of rice seedlings to resist paddy weeds. In our previous work, we successfully elevated the inhibition of culture solutions and leaf extracts of accessions PI and LE by induction of barnyard grass root exudates (Zhang et al. 2018). LE, as a non-allelopathic accession, has been confirmed to possess increased allelopathic potential under induction treatment. Therefore, the aim of this paper is to reveal the differential expression of various genes in the PI and LE rice accessions after treatment with barnyard grass exudates to identify target genes for future research.

## Methods

### Preparation of the barnyard grass root exudates solution

Root exudates of barnyard grass [*Echinochloa crus-galli* (L.) Beauv] was obtained as described in our previous paper (Zhang et al. 2018). In brief, 100 uniform seedlings at the one-leaf stage were cultured in 10 L Hoagland’s solution (Hoagland and Arnon 1950). At the five-leaf stage, the culture solutions were collected and filtered using a Buchner funnel apparatus with suction filtration equipment. The filtrate was concentrated to less than 100 mL by rotary evaporation at 40 °C ± 1 °C and then stored at 4 °C in a refrigerator for 24 h. In this process, the inorganic salts and lower soluble compounds were precipitated and removed by filtration. The filtrate was filtered through a 0.22-μm membrane and diluted to a volume of 100 mL (1 mL concentrate is equivalent to one seedling). This solution was then used as the inducing solution for subsequent experiments.

### Allelopathic induction experiments

The induction experiment of the allelopathic rice accession PI and non-allelopathic rice accession LE was conducted as described in our previous paper (Zhang et al. 2018). Briefly, 50 uniform rice seedlings of each accession at the 1-leaf stage were cultured in 10 L Hoagland’s solution. At the 3-leaf stage, the culture solution was replaced with 5 L Hoagland’s solution (pH 6.0) containing 75 mL of barnyard grass root exudates; these treatments are denoted as PI(+B) and LE(+B). The control treatment consisted of 5 L Hoagland’s solution (pH 6.0) lacking barnyard grass root exudates; these treatments are denoted as PI(−B) and LE(−B). Each treatment had three replicates. After two days of treatment, the roots were harvested and immediately placed into liquid nitrogen and stored at − 80 °C until total RNA extraction. The culture solutions were also collected for inhibitory bioassays and the determination of phenolic acid contents.

### Inhibitory bioassay of rice culture solutions

The culture solutions collected above were filtered using a Buchner funnel apparatus with suction filtration equipment and then filtered through a 0.22-μm membrane. The change in rice allelopathy was estimated based on the inhibitory rate of the rice culture solutions on lettuce (*Lactuca sativa* L.) root length in a laboratory bioassay, as described previously (He et al. 2009; Zhang et al. 2018). Briefly, five germinated lettuce seeds were placed in a 250 mL beaker lined with filter paper, and 5 mL of the test solutions was added. The control consisted of 5 mL of Hoagland’s solution. The beakers were covered with perforated Parafilm and placed into an incubator at 25 °C ± 2 °C with 12 h (8:00–20:00) of 70 μmol m^− 2^ s^− 1^ light intensity. The experiments were replicated four times. After 3 days, the root lengths were measured. The relative inhibition rate (IR) was used to assess the effect of the test solutions compared to the control and was calculated as follows: IR (%) = [(control – treatment)/control] × 100%.

### Determination of phenolic acid concentrations in the rice culture solutions

The contents of seven phenolic allelochemicals in the rice culture solutions, namely, protocatechuic acid, 4-hydroxybenzoic acid, syringic acid, vanillic acid, salicylic acid, ferulic acid, and cinnamic acid, were determined by solid-phase extraction and high-performance liquid chromatography (SPE-HPLC), as described previously (Li et al. 2017). The instrument was an Agilent 1206 HPLC (Agilent Technologies, USA) equipped with a C18 reversed-phase column (ZORBAX SB-C18, 150 mm × 4.6 mm, 5 μm). The mobile phase was a mixture of methanol (A) and 1% phosphoric acid (B) with the following gradient elution program: A:B = 27:73 (9 min), A:B = 30:70 (2 min), and A:B = 50:50 (4 min). The flow rate of the mobile phase was 1.6 mL min^− 1^ and was detected at 280 nm. The injection volume was 5 μL.

### RNA extraction and sequencing

Total RNA was extracted from the roots using TRIzol reagent (Tiangen Bio Co., Ltd., Beijing, China). RNA degradation and contamination were monitored on 1% agarose gels. RNA purity was evaluated using a NanoPhotometer spectrophotometer (IMPLEN, CA, USA). The RNA integrity was assessed using the RNA Nano 6000 Assay Kit for the Agilent 2100 Bioanalyzer (Agilent Technologies, CA, USA). mRNA library preparation and sequencing were out-sourced (Allwegene Tech., Beijing, China). The 12 libraries were sequenced on an Illumina HiSeq 2500 platform, and paired-end 150 bp reads were generated. Reads containing more than 10% poly-N and more than 50% low-quality reads (Q ≤ 20) were removed from the raw data using Trimmomatic v0.33 (Bolger et al. 2014). Concurrently, the Q20 and Q30 values, GC content, and sequence duplication level of the clean data were calculated. All downstream analyses were based on clean, high-quality data.

### Alignment and assembly of the RNA sequencing reads

Since non-allelopathic accession LE is a Japonica Group variety and could have enhanced allelopathic potential under induction treatment (Gealy et al. 2003; Zhang et al. 2018), we chose the Japonica Group as a reference genome annotation so that the results would be more suitable for conventional rice cultivars. RNA-Seq reads were aligned to the rice reference genome (*Oryza sativa* Japonica Group, Ensembl_IRGSP_1.0.34) using TopHat 2 (v2.1.0) (Kim et al. 2013). Each read was mapped with Cufflinks (v2.1.1), which assembled the alignments in Sequence Alignment/Map format into transfrags (Trapnell et al. 2010). The assembly files were then merged with reference transcriptome annotations for further analysis (Trapnell et al. 2012).

### Differential expression analysis

The differential expression of each gene was calculated by quantifying the Illumina reads based on Fragments Per Kilobase of transcript per million fragments mapped (FPKM) (Mortazavi et al. 2008).

$$ \mathrm{FPKM}=\frac{\mathrm{cDNA}\kern0.5em \mathrm{Fragments}}{\mathrm{Mapped}\kern0.5em \mathrm{Fragments}\left(\mathrm{Millions}\right)\times \mathrm{Transcript}\kern0.5em \mathrm{Length}\left(\mathrm{kb}\right)} $$

Pairwise differential expression analyses were performed using the DEGseq package (Anders and Huber 2012) in the R statistical environment (Team 2014). Genes with an FDR-adjusted *P*-value < 0.05 as determined by DEGseq were assigned as differentially expressed. Heatmaps for the differentially expressed genes (DEGs) were constructed using the Pheatmap v. 1.0.8 package (Kolde 2015).

### Functional annotation and classification

Gene Ontology (GO, http://www.geneontology.org/) enrichment analysis of the DEGs was implemented using GOseq (v. 1.22) (Young et al. 2010) using Wallenius’ noncentral hypergeometric distribution, which can adjust for gene length bias in DEGs. Affected pathways were determined using Kyoto Encyclopedia of Genes and Genomes (KEGG, http://www.kegg.jp) (Kanehisa et al. 2008). We used KOBAS (Mao et al. 2005) software to annotate and identify the enriched KEGG pathways of the DEGs.

### Real-time quantitative PCR (RT-qPCR)

The expression patterns of 10 genes associated with secondary metabolite biosynthesis pathways related to rice allelochemicals, including phenolic acids, fatty acids, terpenoids, steroids, and flavones (Mattice et al. 1998; Kim et al. 2000; Kato-Noguchi et al. 2002; Kong et al. 2004b; Macias et al. 2006; Seal et al. 2004; He et al. 2012), were analyzed using RT-qPCR (gene-specific primers are provided in Additional file 1: Table S1). Total RNA was isolated from the roots using TRIzol (Tiangen Bio Co., Ltd., Beijing, China), and cDNA was synthesized using the PrimeScript™ RT reagent Kit with a gDNA Eraser. RT-qPCR was conducted using SYBR® Premix Ex Taq™ II according to the manufacturer’s instructions (Takara, Tokyo, Japan). The actin gene (5-TGTAAGCAACTGGGATGA-3 and 5-CCTTCGTAGATTGGGACT -3) was used as an internal standard. The PCR program was as follows: 30 s of predenaturation at 95 °C, followed by 40 cycles of 5 s at 95 °C and 40 s at 60 °C and steps for dissociation curve generation (10 s at 95 °C, 60 s at 60 °C, and 15 s at 95 °C). The relative gene expression of each target gene was determined using the 2 ^−ΔΔCt^ method (Livak and Schmittgen 2001).

### Statistical analysis

All data were analyzed using DPS (v. 7.05) statistical software (Tang and Feng 2007). Statistical significance was determined using one-way analysis of variance (ANOVA) using least significant difference (LSD) tests at the 5% level of probability.

## Results

### Inhibitory effect of rice culture solutions and phenolic acid contents

**Fig. 1:**
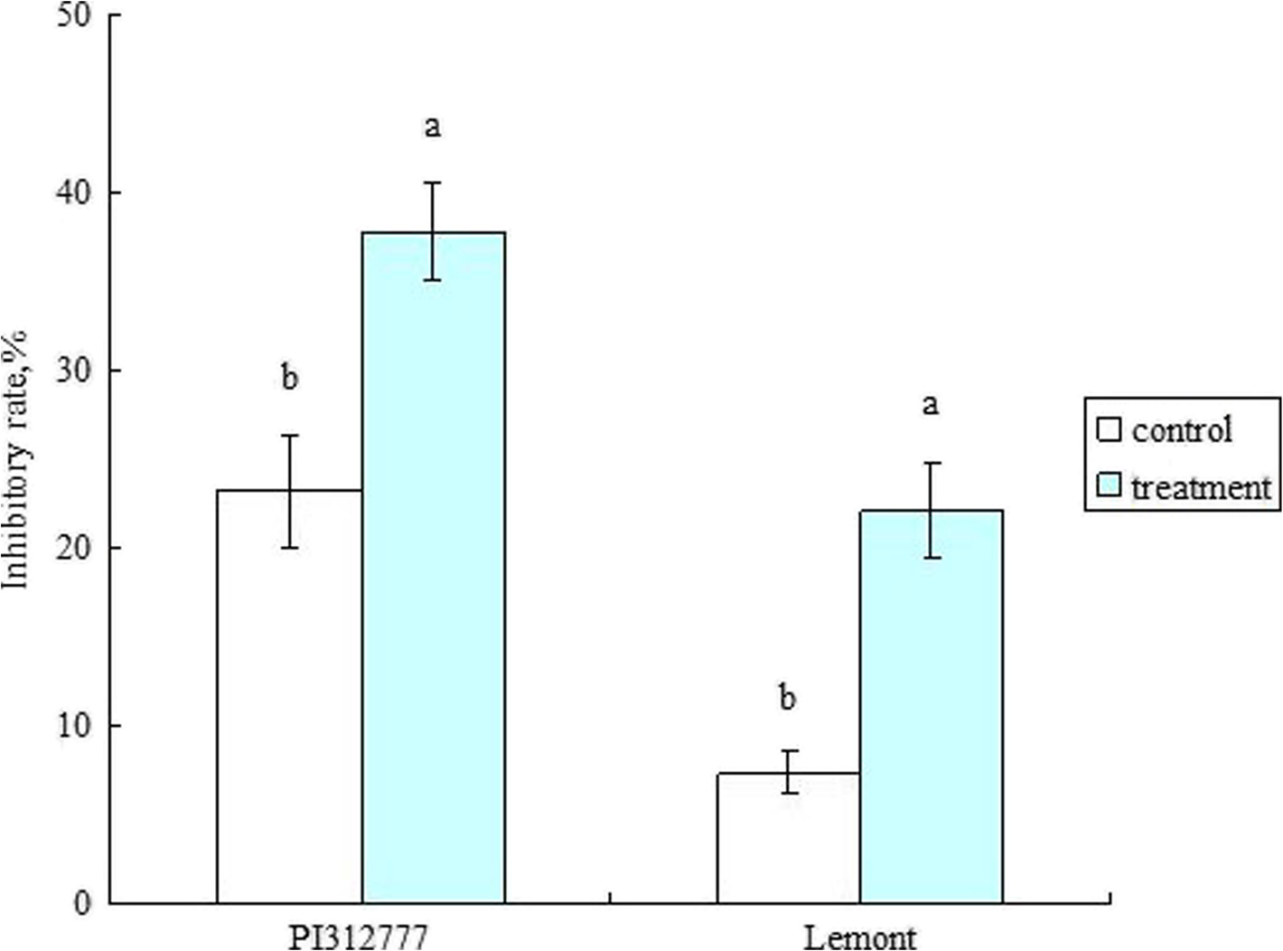
Inhibitory rate of the rice culture solutions on lettuce root length. The bars represent the standard errors of the mean (*n* = 4). Different letters indicate significant differences at *P* < 0.05

Treatment with barnyard grass root exudates significantly increased the inhibitory effect of the two rice culture solutions on lettuce root length (Fig. 1). The inhibitory rates were increased by 14.67% in PI (increased from 23.17% to 37.84%) and 14.75% in LE (increased from 7.34% to 22.09%) compared to the control. The contents of five phenolic allelochemicals were significantly elevated in the PI culture solution. In the LE culture solution, the contents of three phenolic allelochemicals were significantly elevated, while the content of one phenolic allelochemical was significantly decreased (Table 1). The total contents of the seven selected phenolic allelochemicals were significantly higher in both the PI and LE culture solutions than in the control.

**Table 1:** Phenolic acid contents in the rice culture solutions

Phenolic compound (μmol·L^− 1^)	PI312777		Lemont	
	Control	Treatment	Control	Treatment
Protocatechuic acid	0.89 ± 0.12b	1.97 ± 0.10a	0.64 ± 0.07b	0.58 ± 0.05b
4-Hydroxybenzoic acid	0.53 ± 0.04a	0.60 ± 0.07a	0.36 ± 0.09b	0.26 ± 0.04b
Syringic acid	5.20 ± 0.13a	3.64 ± 0.07c	3.17 ± 0.07c	4.35 ± 0.16b
Vanillic acid	0.57 ± 0.01a	0.57 ± 0.07a	0.57 ± 0.04a	0.15 ± 0.03b
4-Coumaric acid	2.86 ± 0.05c	6.26 ± 0.82a	1.58 ± 0.38c	5.45 ± 0.30b
Salicylic acid	8.23 ± 0.61b	14.41 ± 0.17a	6.47 ± 0.21c	9.10 ± 0.28b
Ferulic acid	2.36 ± 0.21b	8.60 ± 0.60a	2.36 ± 0.07b	2.95 ± 0.10b
Total	20.65	36.05	15.16	22.83

Means ± standard errors (SE) of three replicates are indicated. The lowercase letters indicate a significance difference at *P* < 0.05

### Transcriptome sequencing

The transcriptomic responses of the two rice cultivars to barnyard grass root exudates treatment were assayed by RNA sequencing. In total, more than 311 million raw reads from 12 libraries with an average of 25 million reads per condition were obtained (Additional file 2: Table S2). More than 94% high-quality reads with an average Phred quality score of > 30 at each base position were obtained and used for downstream analyses. TopHat was used to map the reference genome. A total of 80.33–79.92% (average 80.11%) of the reads from PI(−B), 80.54–80.16% (average 80.36%) of the reads from PI(+B), 73.82–73.02% (average 73.03%) of the reads from LE(−B), and 81.24–80.10% of the reads (average 80.76%) from LE(+B) were mapped to the reference genome. The percentage of multiple mapped reads was < 2%, and approximately 79% of the reads were uniquely located in the genome (Table 2). In PI(−B), 95.27% of the reads were mapped to exonic regions, 1.36% of the reads were mapped to intronic regions, and 3.37% of the reads were mapped to intergenic regions. In PI(+B), 95.64% of the reads were mapped to exonic regions, 1.05% of the reads were mapped to intronic regions, and 3.31% of the reads were mapped to intergenic regions. In LE(−B), 95.44% of the reads were mapped to exonic regions, 1.31% of the reads were mapped to intronic regions, and 3.26% of the reads were mapped to intergenic regions. In LE(+B), 95.19% of the reads were mapped to exonic regions, 1.31% of the reads were mapped to intronic regions, and 3.51% of the reads were mapped to intergenic regions (Additional file 3: Table S3).

**Table 2:** Analysis of RNA-Seq data from rice roots

	PI(−B)	PI(+B)	LE(−B)	LE(+B)
Total reads	51,505,614	49,158,582	48,516,744	50,824,930
Total mapped reads	41,265,354 (80.11%)	39,508,506 (80.36%)	35,431,373 (73.03%)	41,048,507 (80.76%)
Multiple mapped reads	570,375 (1.11%)	636,262 (1.27%)	740,414 (1.44%)	870,923 (1.71%)
Uniquely mapped reads	40,694,244 (79%)	39,770,017 (79.27%)	36,835,755 (71.8%)	40,620,254 (79.53%)

PI(+B) and LE(+B) represent the PI and LE treatment with barnyard grass root exudate. PI(−B) and LE(−B) represent the corresponding control treatments. The data in parentheses are average values of three replicates

### DEGs specific to exudates treatment

**Fig. 2:**
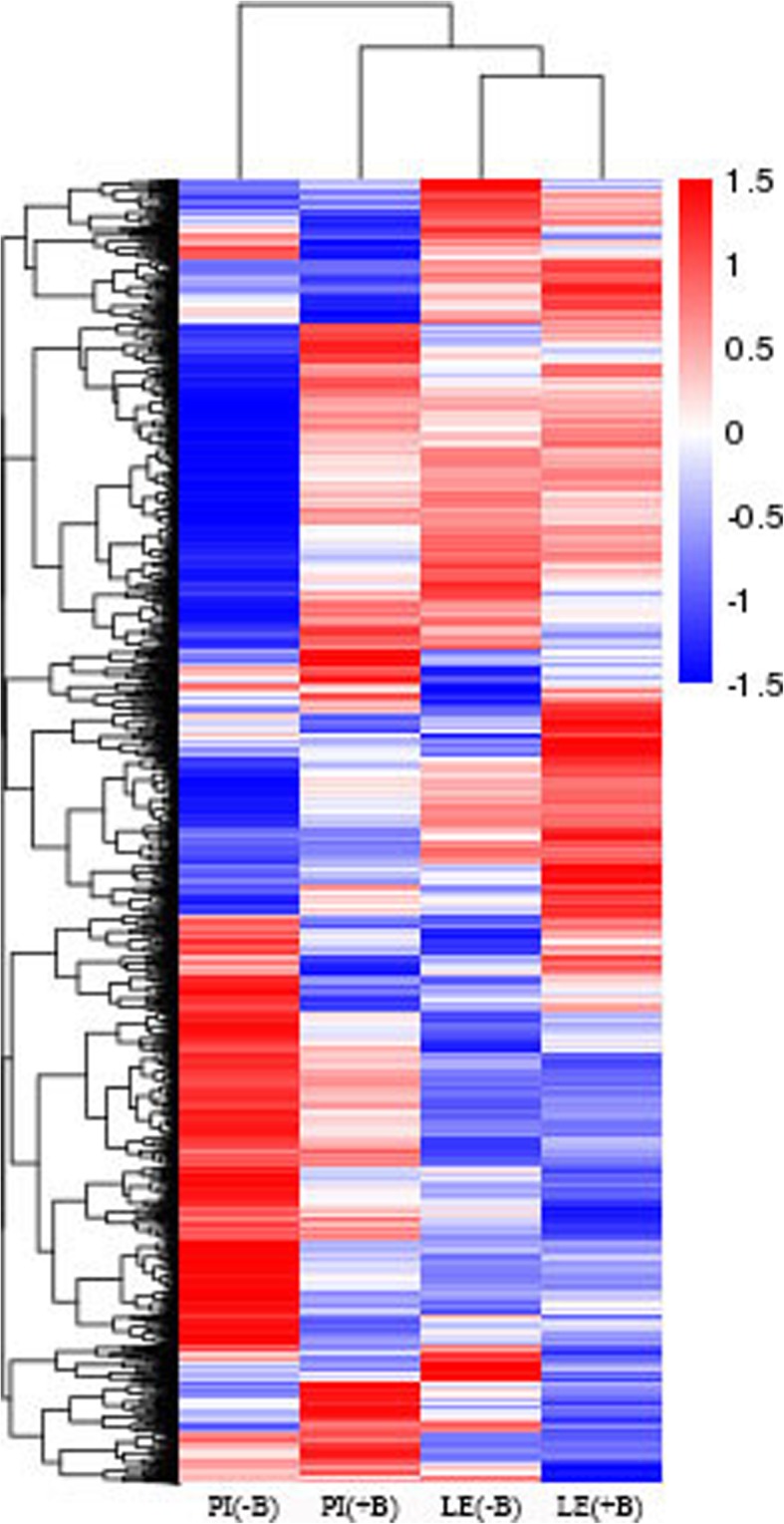
Hierarchical cluster analysis of the DEGs. The color key represents FPKM-normalized log2-transformed counts. PI(+B) and LE(+B) represent the PI and LE accessions treated with barnyard grass root exudates. PI(−B) and LE(−B) represent the corresponding no-induction controls

Gene expression levels were calculated using FPKM. Only the genes with FPKM values > 1 were analyzed. Correlations between the treatments and controls were investigated using cluster analysis in Pheatmap. The gene expression levels of the different rice cultivars varied largely between the treatments and controls (Fig. 2), as indicated by clustering analysis of the various transcripts.

**Fig. 3:**
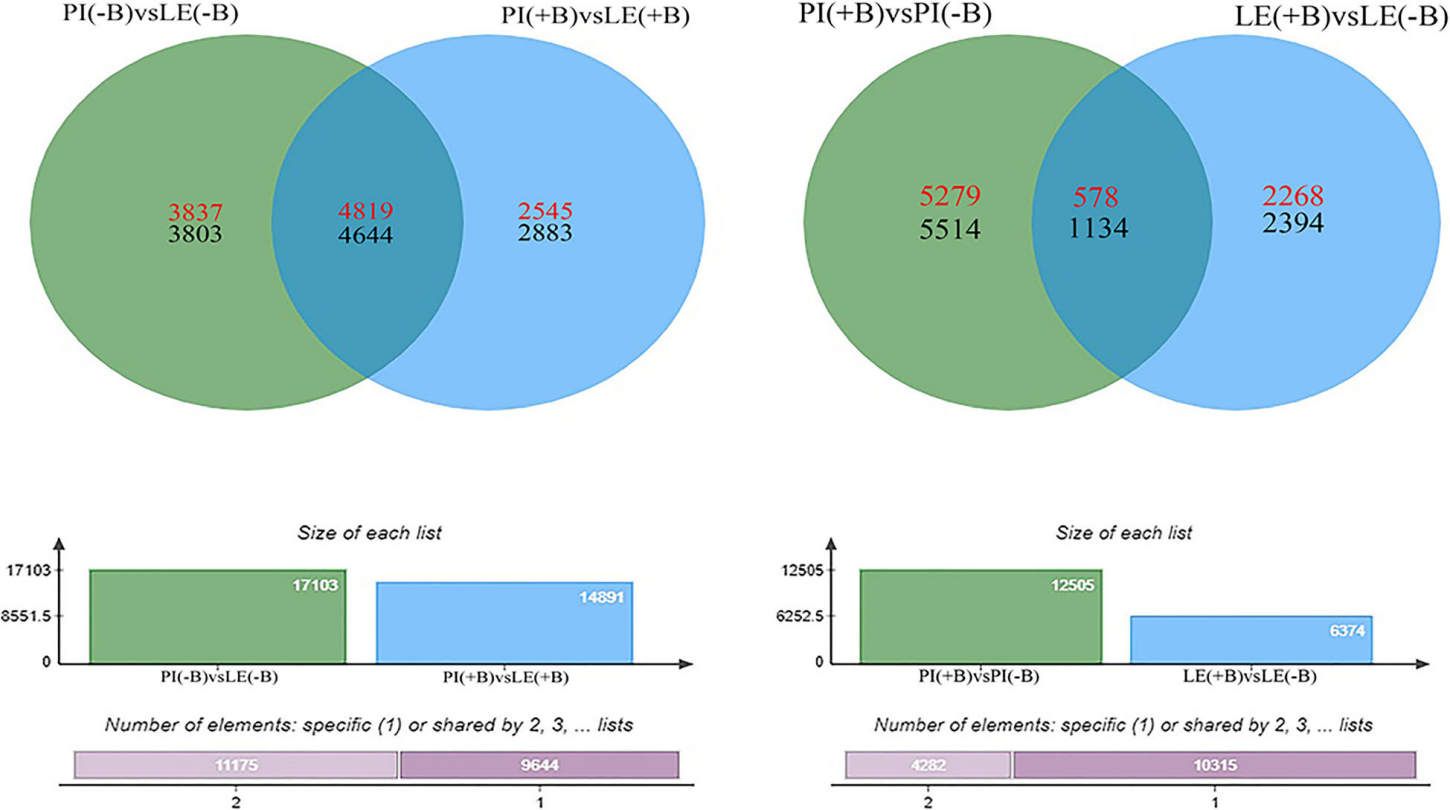
Common and specific DEGs in the two rice cultivars after induction treatment by barnyard grass root exudates. Note: upregulated DEGs are in red, and downregulated genes are in black. PI(+B) and LE(+B) represent the PI and LE accessions treated with barnyard grass root exudates. PI(−B) and LE(−B) represent the corresponding no-induction controls

Comparisons of transcript abundances between PI and LE revealed that 17,103 genes were differentially expressed in PI(−B) vs. LE(−B). Of these, 8656 genes were upregulated, and 8447 genes were downregulated. In addition, 14,891 genes were differentially expressed following root exudates treatment in PI(+B) vs. LE(+B). Of these, 7364 genes were upregulated, and 7527 genes were downregulated (Fig. 3). A total of 4819 upregulated genes and 4644 downregulated genes were shared between PI and LE.

Comparisons of transcript abundance between exudate-treated PI or LE and the controls revealed that 12,505 genes were differentially expressed in PI(+B) vs. PI(−B). Of these, 5857 genes were upregulated and 6648 genes were downregulated. In LE(+B) vs. LE(−B), 6374 genes were differentially expressed following root exudates treatment. Of these, 2846 genes were upregulated, and 3528 genes were downregulated (Fig. 3). A total of 578 upregulated genes and 1134 downregulated genes were shared between PI and LE.

### Functional classification by GO

**Fig. 4:**
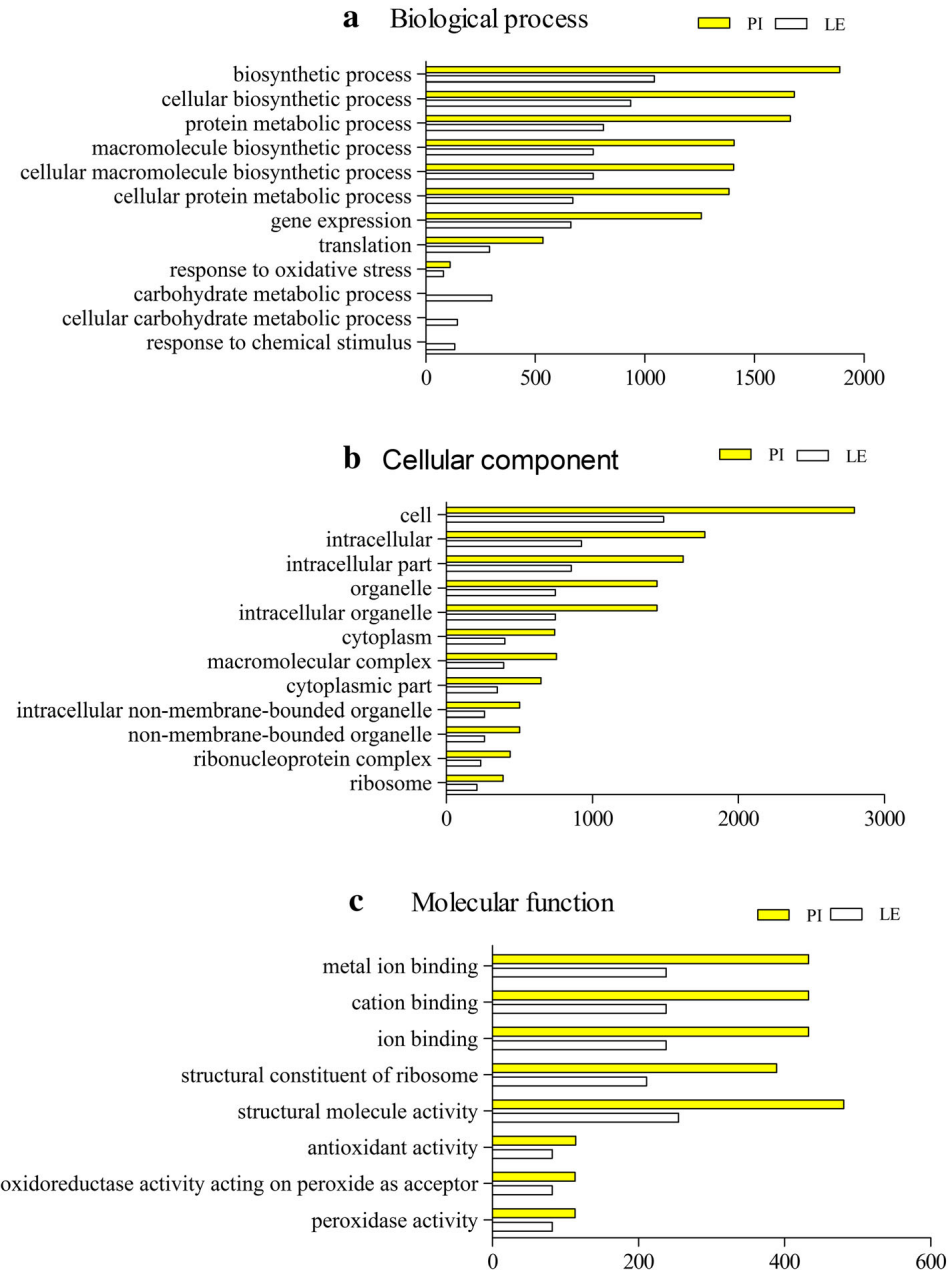
GO annotation clusters of DEGs. GO functional enrichment analysis of DEGs in the roots of different allelopathic rice accessions. Based on sequence homology, 18,879 genes were distributed among the three main categories: **a**, biological process; **b**, cellular component; and **c**, molecular function

Based on the GO analysis, the 12,505 DEGs of PI(+B) vs. PI(−B) and 6374 DEGs of LE(+B) vs. LE(−B) were classified into three categories with 32 functional groups, specifically 12 groups in the biological process category (Fig. 4a), 12 groups in the cellular component category (Fig. 4b), and eight groups in the molecular function category (Fig. 4c).

### GO terms in the biological processes category

We further identified the GO terms in the biological processes category. Fifty and 135 GO terms in this category were significantly upregulated (*P* < 0.05) in LE and PI, respectively (Additional file 4: Table S4 and Additional file 5: Table S5), compared to the corresponding controls. The common GO terms in the two rice accessions included regulation of metabolic process, regulation of primary metabolic process, regulation of cellular biosynthetic process, and regulation of biosynthetic process. Some notable differences in GO terms were also identified between the two rice accessions. For instance, the glucose catabolic process, ATP synthesis coupled electron transport, electron transport chain, amino acid activation, and biosynthetic process terms were highly enriched in PI(+B) compared to PI(−B).

### KEGG pathway mapping and DEGs related to the biosynthesis of secondary metabolites and rice allelochemicals

To identify the metabolic pathways enriched by the DEGs in the two rice accessions, pathway-based analysis was performed using the KEGG pathway database. In PI, 2159 protein sequences were classified into 123 biological pathways, including ketone synthesis and degradation, ribosomes, propionic acid, pantothenic acid metabolism, phenylalanine metabolism and coenzyme A (Co-A) biosynthesis, metabolism of amino acid biosynthesis, and oleic acid. In LE, 1872 gene and protein sequences were classified into 122 significant biological pathways, mainly the citric acid (TCA) cycle, phenylalanine metabolism, terpenoid metabolism, starch and sucrose metabolism, ribosomes, arginine biosynthesis, and metabolism of amino sugars and sugar nucleotides pathways.

In PI(−B) vs. LE(−B) and PI(+B) vs. LE(+B), there were 569 and 529 DEGs, respectively, related to the biosynthesis of secondary metabolites. Specifically, in PI(−B) vs. LE(−B) and PI(+B) vs. LE(+B), 169 and 153 DEGs, respectively, were related to the shikimic acid pathway for phenolic acid synthesis; 177 and 162 DEGs, respectively, were related to the acetic acid pathway for fatty acid production; 94 and 76 DEGs, respectively, were related to the mevalonic acid (MVA) pathway for terpenoid and steroid formation; and 13 and 14 DEGs, respectively, were related to flavonoid synthesis, which involves a combination of the acetic acid and shikimic acid biosynthesis pathways (Table 3). The number of DEGs related to rice allelochemicals between the two treatments were in the order of: acetic acid pathway > shikimic acid pathway > MVA pathway for both PI(−B) vs. LE(−B) and in PI(+B) vs. LE(+B).

**Table 3:** DEGs related to the biosynthesis of secondary metabolites and rice allelochemicals

Description		PI(−B) vs. LE(−B)	PI(+B) vs. LE(+B)	PI(+B) vs. PI(−B)	LE(+B) vs. LE(−B)
Biosynthesis of secondary metabolites		569	529	484	317
Shikimic acid pathway	Phenylpropanoid biosynthesis	86	72	79	64
	Phenylalanine, tyrosine and tryptophan biosynthesis	29	26	25	17
	Phenylalanine metabolism	26	26	27	18
	Tyrosine metabolism	28	29	23	16
	Subtotal	169	153	154	115
Acetic acid pathway	Fatty acid biosynthesis	28	31	23	18
	Biosynthesis of unsaturated fatty acids	20	19	19	12
	Fatty acid metabolism	47	45	40	25
	Fatty acid elongation	14	11	12	5
	Fatty acid degradation	31	26	26	12
	α-Linolenic acid metabolism	30	24	30	16
	Arachidonic acid metabolism	7	6	8	2
	Subtotal	177	162	158	90
Mevalonic acid (MVA) pathway	Terpenoid backbone biosynthesis	32	26	30	18
	Limonene and pinene degradation	4	4	3	1
	Sesquiterpenoid and triterpenoid biosynthesis	4	5	2	2
	Stilbenoid, diarylheptanoid and gingerol biosynthesis	10	11	8	8
	Steroid biosynthesis	17	15	12	12
	Carotenoid biosynthesis	20	14	8	4
	Brassinosteroid biosynthesis	7	1	2	6
	Subtotal	94	76	65	51
Flavonoid biosynthesis		13	14	13	9
	Total	1022	934	874	582

In PI(+B) vs. PI(−B) and LE(+B) vs. LE(−B), there were 484 and 317 DEGs, respectively, related to the biosynthesis of secondary metabolites (Table 3). Specifically, in PI(+B) vs. PI(−B) and LE(+B) vs. LE(−B), 154 and 115 DEGs, respectively, were related to the shikimic acid pathway; 158 and 90 DEGs, respectively, were related to the acetic acid pathway; 65 and 51 DEGs, respectively, were related to the MVA pathway for terpenoid and steroid formation; and 13 and 9 DEGs, respectively, were related to flavonoid synthesis (Table 3). The DEGs between the two treatments were in the order of acetic acid pathway > shikimic acid pathway > MVA pathway in PI(+B) vs. PI(−B) and shikimic acid pathway > acetic acid pathway > MVA pathway in LE(+B) vs. LE(−B).

### Confirmation by RT-qPCR

**Fig. 5:**
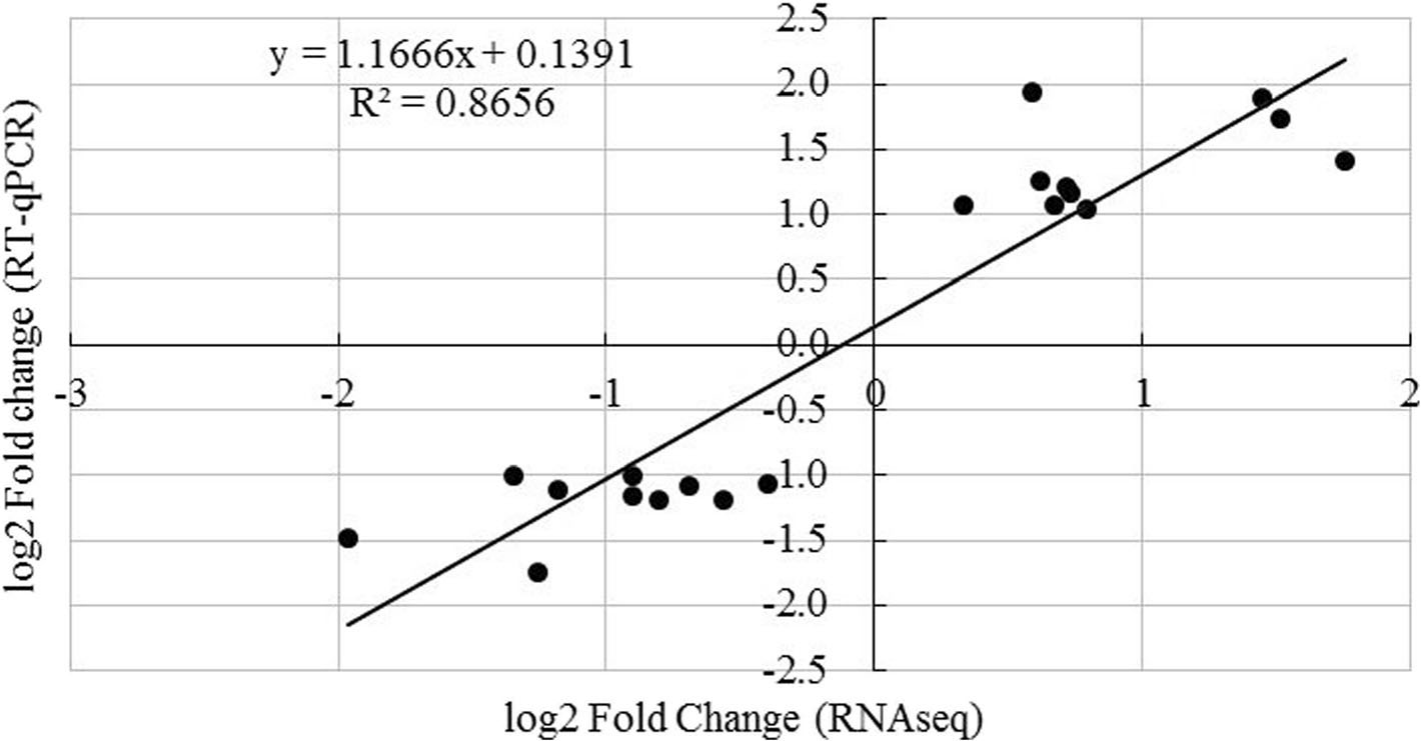
Comparison of the log2-value of fold-change (log2FC) of 10 selected transcripts determined using RNA-Seq and RT-qPCR

RT-qPCR was used to confirm the RNA gene expression patterns of 10 genes related to rice allelochemical biosynthesis (both in PI and LE) and to validate our RNA sequencing results. A good linear correlation was observed between the gene expression fold-changes obtained using RT-qPCR and RNA sequencing (R^2^ = 0.8656) (Fig. 5).

## Discussion

### Increase in rice allelopathy following treatment with barnyard grass root exudates

In this study, treatment with barnyard grass root exudates significantly increased the inhibitory rates of the rice culture solutions on lettuce root growth in both the allelopathic accession PI and non-allelopathic accession LE (Fig. 1). We also discovered that the total contents of phenolic acids previously reported as significant rice allelochemicals were significantly increased in the culture solutions of both PI and LE compared to the controls (Table 1). These results are consistent with our previous findings (Zhang et al. 2018) and once again confirm that induction with barnyard grass root exudates increases rice allelopathy. Although the genetic allelopathy was significantly higher in PI than in LE, the induced allelopathy was roughly equal between the two accessions. This finding further supports that induced allelopathy in rice is an important strategy for enhancing rice allelopathic potential.

### RNA-Seq results

A comparison of the transcript abundances revealed that treatment with root exudates resulted in 17,103 genes that were differentially expressed in PI(−B) vs. LE(−B) and 14,891 genes that were differentially expressed in PI(+B) vs. LE(+B). The total DEGs, number of upregulated DEGs and number of downregulated DEGs in PI(+B) vs. PI(−B) were nearly the same or more than two times greater than those in LE(+B) vs. LE(−B) (Fig. 3). Additionally, 135 and 50 GO terms in the biological processes category were significantly upregulated (*P* < 0.05) in PI(+B) vs. PI(−B) and in LE(+B) vs. LE(−B), respectively (Additional file 4: Table S4, Additional file 5: Table S5). These results indicated that the allelopathic rice accession PI responds more actively than the non-allelopathic rice accession LE to root exudates treatment. Previously reported morphological, physiological, and biochemical results indicated that PI is more sensitive than LE under various biotic and abiotic stresses (Shin et al. 2000; He et al. 2008; Fang et al. 2010; Wang et al. 2010; Kato-Noguchi 2011; He et al. 2012). We suggest that this result derives from the genetic traits of the allelopathic rice accessions.

### DEGs related to the biosynthesis of secondary metabolites and rice allelochemicals

**Fig. 6:**
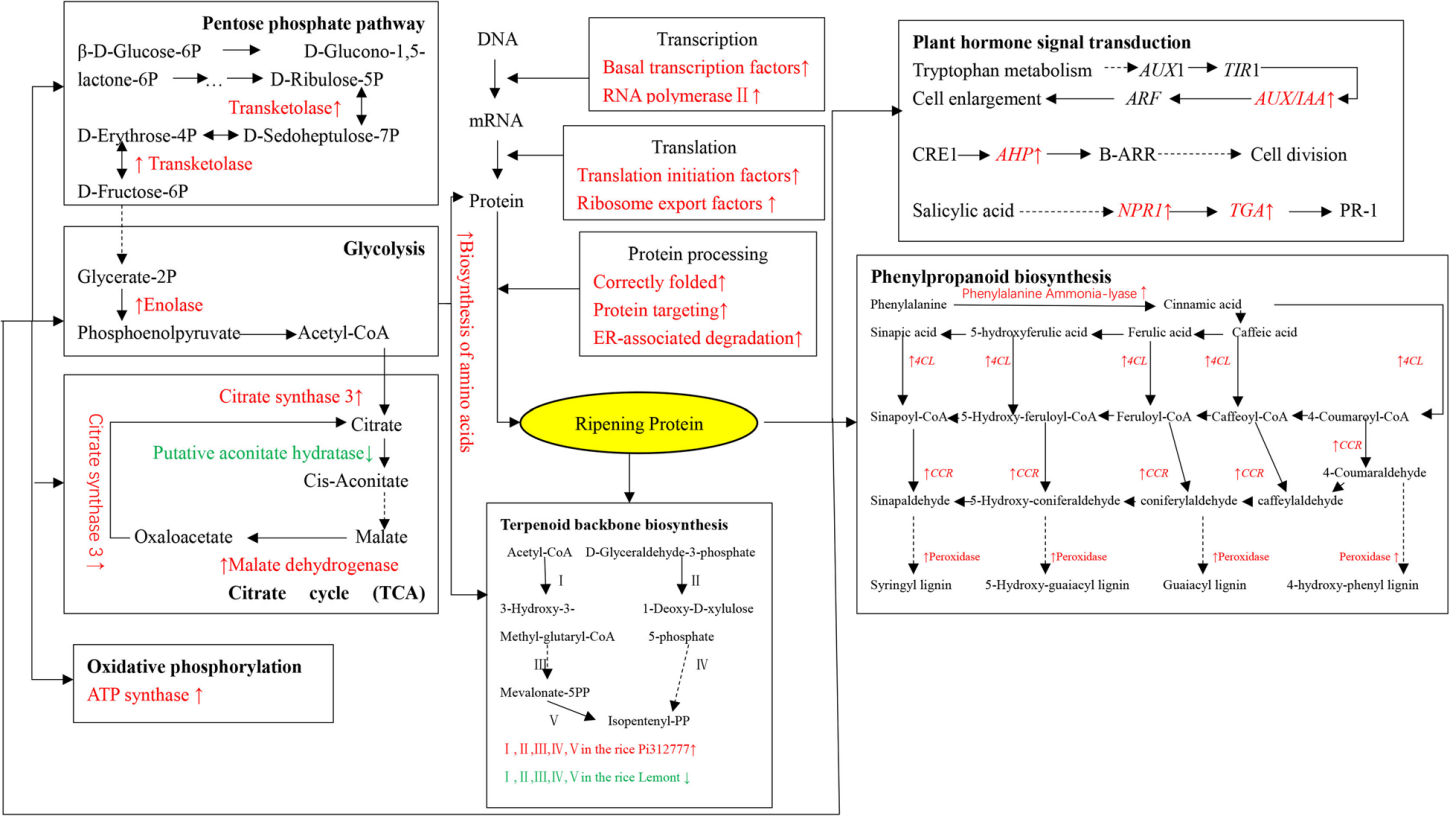
Schematic molecular pathway depicting the changes in the rice root transcriptome under induction treatment by barnyard grass root exudates. The red color represents upregulated genes, and the green color represents downregulated genes. *AUX/IAA*, auxin-responsive protein IAA; *ARF*, auxin response factor; *AHP*, histidine-containing phosphotransfer protein; *NPR*1, regulatory protein NPR1; *TGA*, transcription factor TGA; I: hydroxymethylglutaryl-CoA reductase; II: 1-deoxy-D-xylulose-5-phosphate synthase; III: mevalonate kinase; IV: isopentenyl-diphosphate delta-isomerase; V: diphosphomevalonate decarboxylase; *4CL*, 4-coumarate-CoA ligase; *CCR*, cinnamoyl-CoA reductase

KEGG pathway mapping (Fig. 6) indicated that, under induction treatment, genes related to transcription, such as basal transcription factors and RNA polymerase II, genes related to translation, such as translation initiation factors and ribosome export factors, and genes related to protein modification processes, such as correct folding and ER-associated degradation, were upregulated in both rice accessions. Fukuda et al. (2007) reported that transketolase was upregulated in rice roots exposed to low P, high Al and low pH. The enolase gene of plant roots is usually upregulated under stress conditions such as high salinity, drought, cold and hypoxia (Lal et al. 1998; Forsthoefel et al. 1995). The citrate synthase activity of rye (*Secale cereale* L.) increased by 30% under Al stress (Li et al. 2000). Low temperature caused putative aconitate hydratase downregulation and ATP synthase upregulation in rice leaves and malate dehydrogenase upregulation in rice roots (Yan et al. 2006; Cui et al. 2005; Lee et al. 2009). In our results, transketolase (OS04G0266900), enolase (OS06G0136600), citrate synthase (OS02G0232400), and malate dehydrogenase (OS04G0551200) genes were upregulated, and a putative aconitate hydratase (OS03G0136900) gene was downregulated in both rice accessions under induction treatment. This behavior is consistent with previous reports of rice under stress conditions.

In this study, we are concerned with changes in secondary metabolism related to rice allelochemicals. The rice allelochemicals reported in the current literature are primarily secondary metabolites, such as phenolic acids, fatty acids, terpenoids, steroids, flavones, and hydroxamic acids (Mattice et al. 1998; Kim et al. 2000; Kato-Noguchi et al. 2002; Kong et al. 2004b; Macias et al. 2006; Seal et al. 2004; He et al. 2012; Zhang et al. 2018). Phenolic compounds are synthesized from the shikimic acid pathway; fatty acids are synthesized from the acetic acid pathway; terpenoids and steroids are synthesized from the MVA pathway, and flavonoids are synthesized via a combination of the shikimic acid pathway to supply the C_6_-C_3_ moiety and the acetic acid pathway to supply the C_6_ moiety (Dixon and Paiva 1995; Buchanan et al. 2000). In the present study, the genes related to these three biosynthetic pathways, particularly genes in the shikimic acid and acetic acid pathways, were significantly differentially expressed in both the rice accessions (Table 3). Three genes in the phenylpropanoid biosynthesis pathway were upregulated in both rice accessions under induction treatment; however, five genes in the terpenoid biosynthesis pathway were upregulated in PI and downregulated in LE. Moreover, some genes in the plant hormone signal transduction pathway, such as auxin-responsive protein IAA (*AUX/IAA*) and histidine-containing phosphotransfer protein (*AHP*), which are related to cell enlargement and cell division, and regulatory protein NPR1 (*NPR*1) and transcription factor TGA (*TGA*), which are related to salicylic acid, were also upregulated after induction treatment (Fig. 6). These findings suggest that the response of rice to barnyard grass root exudates treatment involves physiological and biochemical processes, as well as biosynthesis pathways of secondary metabolites. Phenolic acids, fatty acids, flavonoids, and terpenoids, which are the downstream metabolites of the shikimic acid, acetic acid and MVA pathways, were significantly expressed in rice under barnyard grass root exudates treatment and further resulted in changes in rice allelopathy. Recently, publications have reported that potential signaling chemicals of barnyard grass root exudates are benzoxazinoids, (−)-loliolide and jasmonic acid (Guo et al. 2017; Kong et al. 2018). Increasing the allelopathic potential of conventional cultivars by the induction method may be a promising and practicable approach in the application of rice allelopathy.

### Phenolic acids and the associated biosynthesis pathways

Phenolic acids constitute one type of rice allelochemical. In contrast to the findings of Olofsdotter et al. (2002), who miscalculated the importance of phenolic acids in allelopathy, our previous report found that phenolic acids constitute significant allelochemicals in rice (Li et al. 2017). Phenolic acids are synthesized from the conversion of phenylalanine and tyrosine to phenylpropanoid (C_6_-C_3_) intermediates (Herbert 1989; Dixon and Paiva 1995; Buchanan et al. 2000; Crozier et al. 2006). Shikimic acid is a key intermediate in the biosynthesis of phenylalanine and tyrosine, and the enzyme phenylalanine ammonia lyase (PAL) is an initial key enzyme in the biosynthesis of phenolic compounds and catalyzes the conversion of phenylalanine to cinnamic acid and other subsequent phenolic acids (Dixon and Paiva 1995; Buchanan et al. 2000). Another downstream function of phenylalanine and cinnamic acid is supplying the C_6_-C_3_ moiety of flavonoids (Herbert 1989; Dixon and Paiva 1995; Buchanan et al. 2000; Crozier et al. 2006). Genes related to the shikimic acid pathway, such as genes associated with phenylpropanoid biosynthesis, phenylalanine metabolism, and phenylalanine and tyrosine biosynthesis, were significantly differentially expressed in both PI and LE compared to the control (Table 3). PAL, 4-coumarate-CoA ligase (*4CL*), and cinnamoyl-CoA reductase (*CCR*) were upregulated in both rice accessions under induction treatment (Fig. 6). *PAL* is always upregulated in PI and LE upon exposure to stress conditions, such as N, P, K, UV-B, and water stress (Shin et al. 2000; He et al. 2008; Fang et al. 2010; Wang et al. 2010). In a previous study, the roots of PI and LE rice co-cultured with barnyard grass at a ratio of 1:1 exhibited 5.06-fold and 2.24-fold up-regulation of *PAL*, respectively, compared to mono-cultured controls; furthermore, the contents of phenolic allelochemicals were 3.97 times and 1.5 times higher in the PI and LE co-cultured with barnyard grass, respectively, than in the mono-cultured controls (He et al. 2012). Our previous results demonstrated that *PAL* in PI and LE was upregulated in a hydroponic system in response to barnyard grass root exudates treatment, and this upregulation was accompanied by increases in the phenolic acids in the rice leaves and roots and the culture solution (Zhang et al. 2018). Fang et al. (2013, 2015) provided additional evidence that barnyard grass growth inhibition was significantly higher in a *PAL*-overexpression line of PI and significantly lower in a *PAL*-RNA interference line of PI compared to a PI control under identical conditions. It is thus evident that *PAL* and phenolic acids contribute significantly to the observed increase in rice allelopathy under barnyard grass root exudates treatment.

## Conclusions

The allelopathic potential of both allelopathic accession PI and non-allelopathic accession LE could be significantly enhanced by application of barnyard grass root exudates in a hydroponic system. Genes related to the biosynthesis pathways of reported rice allelochemicals were significantly differentially expressed in both rice accessions under barnyard grass root exudates treatment. *PAL* demonstrates great potential for applications in the regulation of chemical induction.

## Additional files

Additional file 1: **Table S1.** Primer (XLSX 10 kb)

Additional file 2: **Table S2.** Sequencing data (XLSX 9 kb)

Additional file 3: **Table S3.** Number of mapped reads (XLSX 9 kb)

Additional file 4: **Table S4.** Up-regulated genes in LE(+B) compared with LE(−B). GO terms for biological processes enriched with a *P* < 0.05 are provided. (XLSX 11 kb)

Additional file 5: **Table S5.** Up-regulated genes in PI(+B) vs. PI(−B). GO terms for biological processes enriched with *P* < 0.05 are provided. (XLSX 16 kb)

## Abbreviations

DEGs: Differentially expressed genes
KEGG: Kyoto Encyclopedia of Genes and Genomes
KO: KEGG Orthology
LE: Non-allelopathic rice accession Lemont
LE(+B): The non-allelopathic accession LE treated with barnyard grass root exudates
LE(−B): The non-allelopathic accession LE no induction controls
MVA: Mevalonic acid
PAL: Phenylalanine ammonia lyase
PI: Allelopathic rice accession PI312777
PI(+B): The allelopathic accession PI treated with barnyard grass root exudates
PI(−B): The allelopathic accession PI no induction controls
RNA-Seq: RNA sequencing
RT-qPCR: Real-time quantitative polymerase chain reaction
SPE-HPLC: Solid-phase extraction and high-performance liquid chromatography
